# Evaluation of ophthalmic vascular and neuroretinal alterations in fibromyalgia syndrome: a cross-sectional comparative study

**DOI:** 10.1007/s00296-024-05662-w

**Published:** 2024-07-16

**Authors:** Gülşah Yaşa Öztürk, Duygu Topaktaş Emekli, Eda Sahutoglu, Burhan Fatih Kocyigit

**Affiliations:** 1 Faculty of Medicine, Department of Physical Medicine and Rehabilitation, University of Health Sciences, Adana City Training and Research Hospital, Adana, Türkiye; 2 Department of Ophthalmology, Adana City Training and Research Hospital, Adana, Türkiye

**Keywords:** Fibromyalgia, Retina, Angiography, Optical coherence tomography, Optical imaging

## Abstract

**Introduction:**

Fibromyalgia syndrome (FMS) is a prevalent rheumatic disorder, and its pathogenesis includes genetic, neuroendocrine, and autonomic abnormalities, which may impact ocular structures. The aim was to conduct a comparative analysis of the ophthalmic vasculature and the retinal nerve fiber layer (RNFL) thickness between FMS and control groups using optical coherence tomography (OCT) and OCT angiography (OCTA).

**Methods:**

This cross-sectional comparative study included 43 FMS patients and 40 healthy controls recruited from a tertiary education and research hospital between January 2024 and May 2024. All patients satisfied the 2016 American College of Rheumatology criteria for FMS and consented. OCT and OCTA were used to assess the RNFL thickness and the retinal microvasculature structure. The Fibromyalgia Impact Questionnaire (FIQ) was performed to evaluate disease severity.

**Results:**

The study found significantly higher total retinal parafoveal thickness and foveal density in FMS patients (*p* = 0.017 and *p* = 0.044, respectively). Nevertheless, there were no significant differences among the groups concerning total retinal foveal thickness, foveal avascular zone characteristics, superficial and deep capillary plexus densities, choriocapillaris flow area, and outer retinal flow area values (*p* > 0.05). The RNFL thickness in all quadrants did not reveal significant differences between the groups (*p* > 0.05). Furthermore, there was no significant correlation between FIQ scores and OCTA parameters or RNFL thickness values (*p* > 0.05).

**Conclusion:**

The study revealed slight differences in retinal parafoveal thickness and foveal density in FMS patients, but no substantial vascular or neurodegenerative alterations were observed compared to healthy controls. These data indicate that FMS may not substantially affect ocular structures, contrary to earlier hypotheses.

**Supplementary Information:**

The online version contains supplementary material available at 10.1007/s00296-024-05662-w.

## Introduction

The core of fibromyalgia syndrome (FMS), a rheumatic condition, is widespread chronic musculoskeletal pain. Other cognitive and physical signs include fatigue, sleep disorders, brain fog, depression, and anxiety [1, 2]. This clinical picture stays with many patients for years, resulting in regular trips to healthcare centers. FMS and its symptoms can be disabling and devastating for specific individuals [3]. FMS is a prevalent rheumatic disorder that ranks second in frequency, with a preponderance of female cases. The disease primarily impacts individuals between 20 and 60 [4].

Despite our limited understanding of the pathophysiology of FMS, several aspects of the process have been identified. Genetics, stressful circumstances, neuroendocrine defects, and irregularities of the central nervous and autonomic systems are among the factors accelerating the course of disease [5–7].

Research on the hypothalamic-pituitary-adrenal axis and the system of autonomic neurons has demonstrated that inappropriate sympathetic excessive activity can cause impaired regulation of blood pressure, hasten the development of endothelial dysfunction and vessel wall sclerosis, resulting in narrowing of blood vessels and reduced blood flow to the choroid, a condition known as choroidal ischemia [8–10].

In recent years, ophthalmology has benefited from the introduction of advanced digital imagery innovations, which provided professionals with additional parameters to diagnose and monitor diseases. Optical coherence tomography (OCT) enables professionals to accurately assess the neuroretinal layer thickness [11]. OCT angiography (OCTA) visualizes the microvascular architecture of the retina and choroid by detecting the signals emitted by red blood cells in these regions. This diagnostic tool is beneficial for identifying ocular vascular conditions such as retinopathy due to diabetes, embolism, and glaucoma [12].

While it has been proposed that FMS is a condition linked to alterations in central nervous system function, vessels, and structure, it is also plausible that structural abnormalities impacting the neuroretina may be observed in individuals with FMS [13]. This study aimed to assess the ophthalmic vasculature of FMS patients and compare data with healthy individuals. In order to achieve this objective, the superficial-deep vascular systems in the retina, as well as the choriocapillaris vessels and retinal nerve fiber layer (RNFL), were examined utilizing OCT and OCTA.

## Methods

This is a cross-sectional and comparative study carried out in a tertiary education and research hospital’s Physical Medicine and Rehabilitation and Ophthalmology clinics. All patients in the study were diagnosed at the outpatient clinic for physical medicine and rehabilitation. The date range in which the article was conducted is January 2024 and May 2024. Only individuals who satisfied the 2016 American College of Rheumatology (ACR) criteria were enrolled in the FMS group [14]. The study comprised individuals over 18 who consented to participate and involved healthy controls aligned with the patient group’s baseline characteristics. Newly diagnosed individuals were included in the study, whereas those receiving FMS-related drugs were excluded. Exclusion criteria comprise dementia, glaucoma, retinal disorders, uveitis, iridocyclitis, cardiovascular diseases, hypertension, diabetes mellitus, inflammatory rheumatic diseases, autoimmune diseases, vasculitis, neurological diseases, prior ophthalmic operations, and drug use that can have an impact on outcomes. All participants underwent routine blood tests. Individuals who did not fulfill the specific requirements for the research were excluded from participating in the study. An evaluation was conducted on the hospital registry system to ascertain whether the individuals fulfilled the exclusion criteria. The Ministry of Health system was utilized to authenticate prescription medications.

Data on the participants’ age, sex, body mass index (BMI), duration of symptoms, occupational status, and educational status were obtained. The same physical medicine and rehabilitation specialist and ophthalmologist conducted the clinical screenings, OCT and OCTA measurements, and further examinations. The Fibromyalgia Impact Questionnaire (FIQ) was used to evaluate the severity of the condition.

### Fibromyalgia impact questionnaire

The FIQ is a thorough instrument specifically developed to evaluate the entire influence of FMS on a patient’s daily life. The FIQ was initially created by Burckhardt et al. [15] in 1991 and has gained widespread acceptance as a standard tool in clinical and research environments for assessing FMS severity. It has been demonstrated that the Turkish version of the FIQ is reliable and valid [16]. There are ten fundamental questions included in the questionnaire. A score of 100 is the highest possible, and higher values indicate a more severe health issue.

### Optical coherence tomography and retinal nerve fiber layer assessment

An automatic computer algorithm determines RNFL thickness without needing a user or reference plane. The measurements are displayed as a thickness map according to the position of the RNFL around the optic disc: 12 clock quadrants, four quadrants, and the average RNFL thickness. For these measurements to be reliable, the 3.4 mm diameter circle must be placed around the optic disc equidistant from all quadrants. In the newer systems, the device automatically provides the ring placement without user intervention.

In SD-OCT devices, numerical data are given in disc area, mean cup volume, vertical and mean C/D ratios, RNFL quadrant thickness, mean RNFL thickness, and neuroretinal rim area. These data are compared with normative data prepared according to racial variation and age in the device memory, and the deviations from the parameters considered normal are shown with color-coded maps. White and green bands indicate typical values, and yellow borderline and red abnormal values. In the healthy population, 5% are in the white band above the green, 90% in the green band, 4% in the yellow band, and 1% in the red band [17].

After achieving mydriasis, the OCT-RNFL test was evaluated with Zeiss Cirrus HD-OCT Model 4000 using an Optic Disc Cube 200 × 200 module. Care was taken to ensure that the signal strength was more than 5/10. Mean retinal nerve fiber thickness (in µm) on the Optic Disc Cube 200 × 200 module and nerve fiber thickness in 4 quadrants were taken as the basis.

### Optical coherence tomography angiography

OCTA is a noninvasive new-generation imaging system that provides detailed visualization of vascular structures by detecting the motion contrast of blood in the posterior segment vessels without using fluorescein. The first step in obtaining the image on the OCTA device is detecting motion contrast. Secondly, consecutive B-scans are taken, and the differences between the images are detected. In the last step, this information is processed by the software program to create an image of the retinal vascular structures. Meanwhile, the device software corrects motion artifacts caused by eye movements [18].

The OCTA device detects motion contrast in the posterior segment tissues and calculates the signal difference between static and nonstatic tissues. This creates three-dimensional image cubes from the en-face images of the retina, choroid, and optic nerve. The device’s segmentation feature allows for analyzing these image cubes in layers.

The most common scan sizes in OCTA are 3 × 3 mm and 6 × 6 mm. The detail decreases as the examined area increases. In our study, after sufficient mydriasis was achieved in the patients, the same personnel performed OCTA measurements using an AngioVue (Optovue Inc, Fremont, CA, USA) SD-OCT (spectral domain optical coherence tomography) device. The wavelength used in the device is 840 nm, axial resolution is 5 microns, transverse resolution is 15 microns, and scan rate (A scan/sec) is 70 kHz. All participants were evaluated with 3 × 3 images and OCTA images with scan quality below 8; segmentation artifacts, motion artifacts, and projection artifacts were excluded from the study.

Foveal avascular zone area (mm^2^), foveal avascular zone perimeter (mm), and foveal density (%) were measured automatically with the device’s phase assessment tool. Density assessment tool measured vascular density (%) in the area (fovea, parafovea) divided by circles of 1 mm and 3 mm diameter with foveal avascular zone as the center of superficial and deep capillary plexus. Each segment was divided into four equal quadrants: temporal, superior, nasal, and inferior. With the flow assessment tool, blood flow in the outer retina and choriocapillaris was recorded in mm^2^ in an area with a central radius of 1 mm and an area of 3.142 mm^2^.

**Fig. 1:**
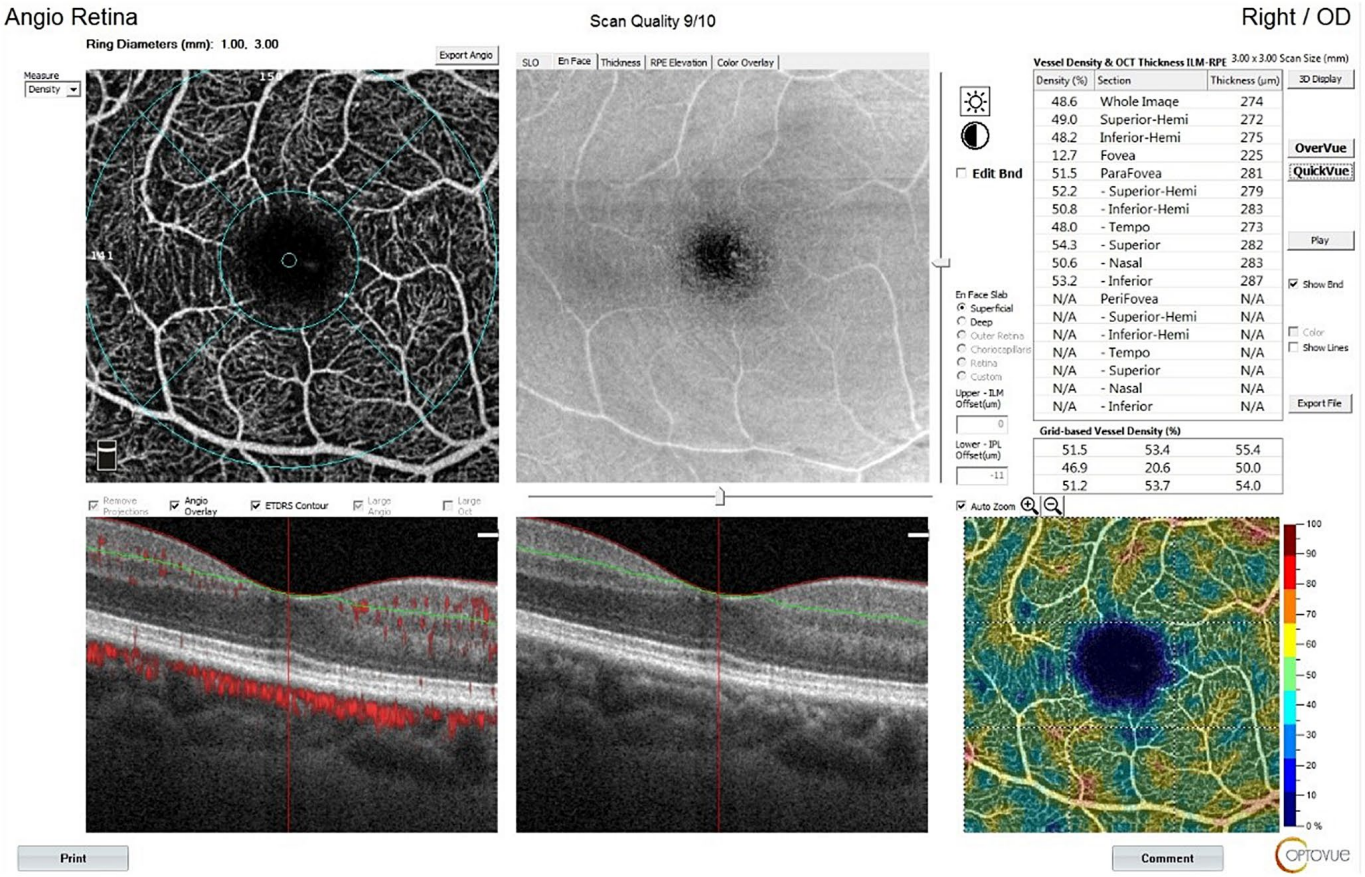
Superficial vascular density assessment using optical coherence tomography angiography

**Fig. 2:**
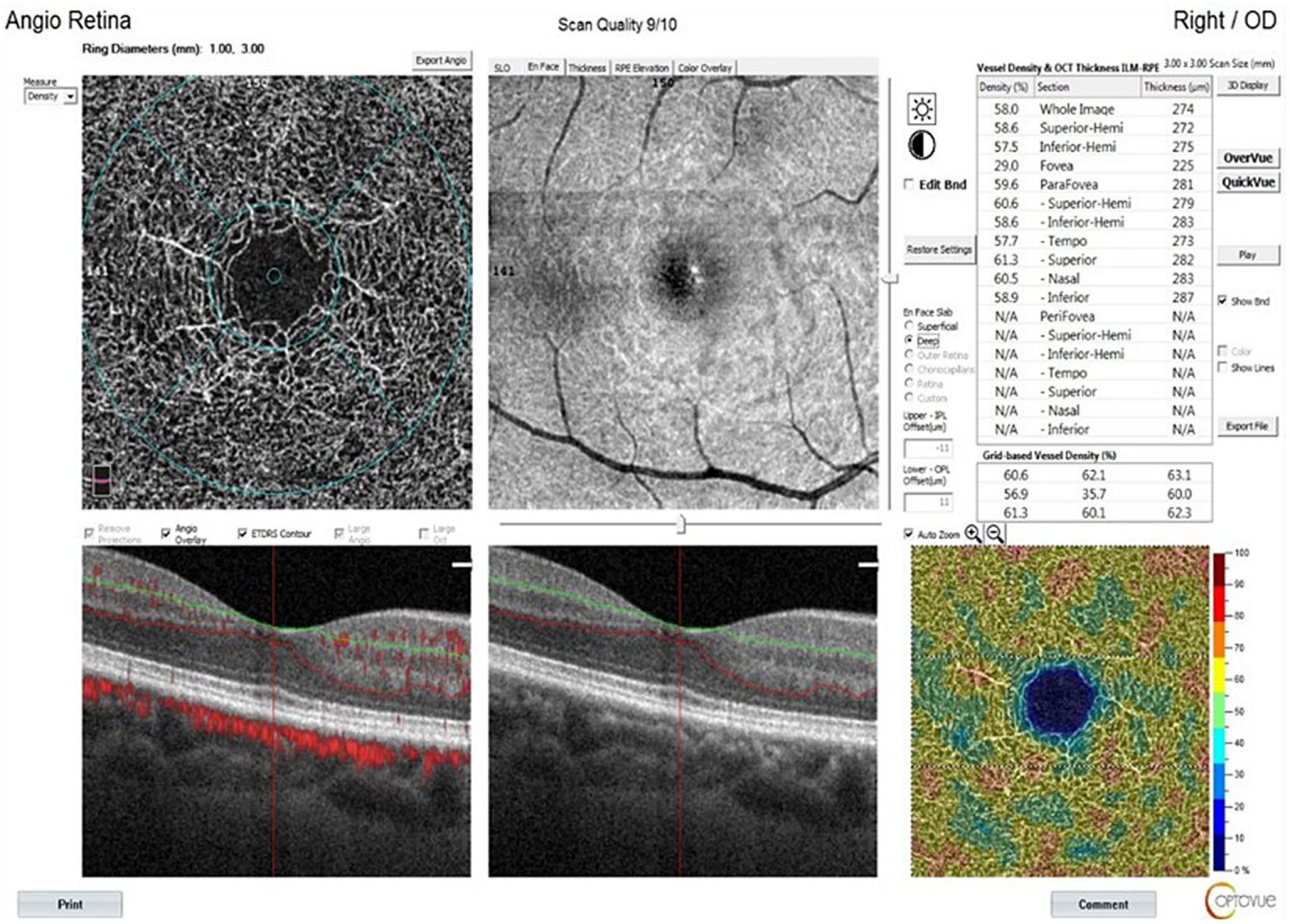
Deep vascular density assessment using optical coherence tomography angiography

**Fig. 3:**
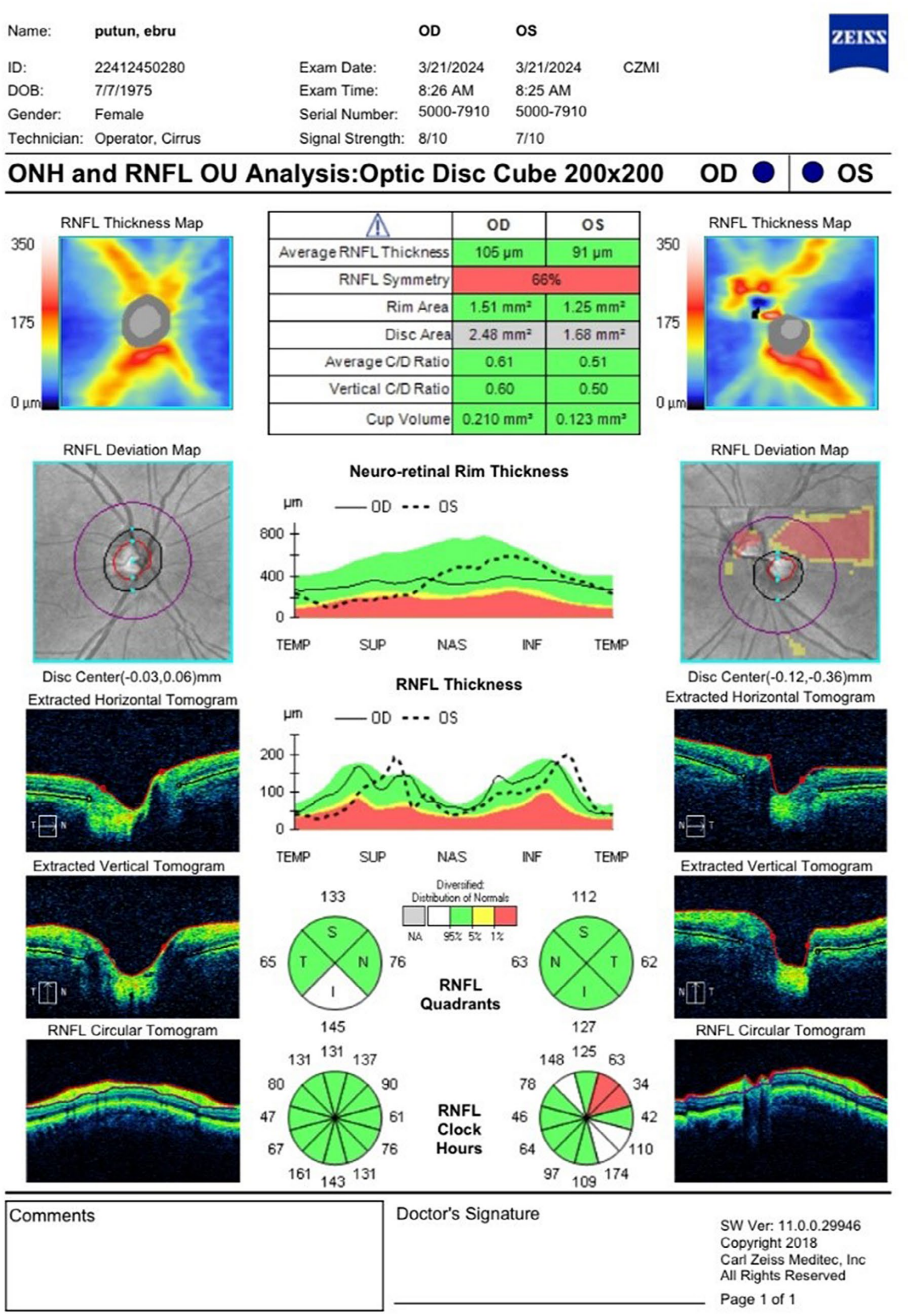
Retinal nerve fiber layer thickness assessment

Measures were conducted for both eyes; however, only the data from the right eye were used in the statistical analysis. OCTA and RNFL assessments are visualized in Figs. 1, 2 and 3.

The local Research Ethics Committee approved this study after thoroughly evaluating the ethical considerations (approval date: 07.12. 2023 and number: 2999). Additionally, written consent was obtained from all participants. The Helsinki principles were followed consistently throughout the entire investigation.

### Statistical analysis

Data analysis was performed using SPSS vn 23 software. Results were presented as a number, percentage, mean ± standard deviation, and median (minimum-maximum). The decision on the conformity of the data to normal distribution was based on the results of the Shapiro-Wilk test. Continuous variables were compared between groups using the Independent Sample t-test and the Mann-Whitney U-test. The chi-square test was also used for categorical variable comparisons. Correlations were evaluated using Pearson and Spearman rho tests. *p* < 0.05 was considered statistically significant.

## Results

Initial enrollment included 45 FMS patients and 45 healthy controls who satisfied the study’s standards. Two FMS patients and four healthy controls did not comply with ophthalmologic examination. Additionally, one healthy control had suspicious OCTA measurements. Following the exclusion of these subjects, the research contained 43 FMS patients and 40 healthy controls. The mean ages of FMS patients and healthy controls were 49.51 ± 9.52 and 52.78 ± 10.88, respectively. The mean BMI of FMS patients was 25.41 ± 3.12, while 25.60 ± 3.85 in healthy controls. Thirty-eight (88.4%) of the FMS patients and 30 (75%) healthy controls were female. There were no significant disparities in the fundamental features of groups (*p* > 0.05) (Table 1).

**Table 1:** The baseline features of the fibromyalgia and control groups

	FMS group	Control group	*p*
**Age** ^*^ **(years)**	49.51 ± 9.52	52.78 ± 10.88	0.149
**BMI** ^*^ **(kg/m²)**	25.41 ± 3.12	25.60 ± 3.85	0.798
**Sex (n) (%)**			
**Female**	38 (88.4)	30 (75)	0.114
**Male**	5 (11.6)	10 (25)	
**Educational status (n) (%)**			
**Primary School**	18 (41.9)	11 (27.5)	0.101
**Middle School**	9 (20.9)	3 (7.5)	
**High School**	12 (27.9)	16 (40)	
**University**	3 (7)	7 (17.5)	
**Higher than university**	1 (2.3)	3 (7.5)	
**Occupational status (n) (%)**			
**Working**	4 (9.3)	11 (27.5)	0.063
**Not working/Housewife**	32 (74.4)	21 (52.5)	
**Retired**	7 (16.3)	8 (20)	

^*^: Data are expressed as mean ± standard deviation

BMI: Body mass index; FMS: Fibromyalgia syndrome; kg: Kilogram; m²: Square meter; n: Number; %: Percentage

The groups were compared based on OCTA characteristics. The FMS group exhibited significantly higher total retinal parafoveal thickness and foveal density (*p* = 0.017 and *p* = 0.044, respectively). However, there were no significant differences between the groups in terms of total retinal foveal thickness, foveal avascular zone area, foveal avascular zone perimeter, superficial capillary plexus foveal vascular density, superficial capillary plexus parafoveal vascular density (including subfractions), deep capillary plexus foveal vascular density, deep capillary plexus parafoveal vascular density (including subfractions), choriocapillaris flow area and outer retinal flow area values (*p* > 0.05) (Table 2).

**Table 2:** Comparison of optical coherence tomography angiography parameters between fibromyalgia and control groups

Total retinal foveal thickness^*^ (µm)	214.42 ± 15.81	210.38 ± 18.38	0.285
Total retinal parafoveal thickness^*^ (µm)	282.30 ± 12.04	275.15 ± 14.74	0.017
Foveal avascular zone area^a^ (mm²)	0.29 (0.09–1.01)	0.34 (0.08–0.61)	0.172
Foveal avascular zone perimeter^a^ (mm)	2.16 (1.22–5.08)	2.26 (1.11–3.39)	0.245
Foveal density^a^ (%)	52.03 (30.83–58.45)	50.83 (37.38–58.77)	0.044
SCP foveal vascular density*(%)	49.40 ± 4.67	48.08 ± 4.74	0.205
SCP parafoveal vascular density^a^ (%)	50.80 (31.4–57.7)	49.40 (36.1–53.4)	0.234
SCP parafoveal temporal vascular density^a^ (%)	48.80 (30.6–55.5)	47.50 (31.6–53.2)	0.290
SCP parafoveal superior vascular density^a^ (%)	51 (2.9–59.4)	51.65 (36.2–55.4)	0.535
SCP parafoveal nasal vascular density^a^ (%)	49.3 (30.2–57.4)	48.1 (29.6–52.8)	0.245
SCP parafoveal inferior vascular density^a^ (%)	52.10 (28.4–60.7)	50.80 (36.8–55.4)	0.497
DCP foveal vascular density^*^ (%)	32.55 ± 7.27	31.01 ± 7.91	0.360
DCP parafoveal vascular density^*^ (%)	55.98 ± 4.04	55.32 ± 3.31	0.422
DCP parafoveal temporal vascular density^*^ (%)	56.11 ± 3.87	55.69 ± 2.88	0.578
DCP parafoveal superior vascular density^*^ (%)	56.01 ± 4.72	55.15 ± 4.53	0.401
DCP parafoveal nasal vascular density^a^ (%)	57.3 (44.7–67.4)	54.95 (31.3–62.1)	0.299
DCP parafoveal inferior vascular density^*^ (%)	55.47 ± 4.57	55.85 ± 3.42	0.671
Choriocapillaris flow area^*^ (mm²)	2.11 ± 0.11	2.12 ± 0.14	0.731

^*^: Data are expressed as mean ± standard deviation; ^a^: Data are expressed as median (minimum - maximum)

SCP: Superficial capillary plexus; DPC: Deep capillary plexus; FMS: Fibromyalgia syndrome

There was no significant difference between the groups in average RNFL thickness, temporal RNFL thickness, superior RNFL thickness, nasal RNFL thickness, and inferior RNFL thickness measurements (*p* > 0.05) (Table 3).

**Table 3:** Comparison of retinal nerve fiber layer parameters between fibromyalgia and control groups

Parameter^*^	FMS group	Control group	*p*
Average RNFL thickness (µm)	93.09 ± 9.39	91.83 ± 6.49	0.480
Temporal RNFL thickness (µm)	64.74 ± 9.13	66 ± 7.87	0.506
Superior RNFL thickness (µm)	114.40 ± 16.23	111.55 ± 13.61	0.391
Nasal RNFL thickness (µm)	68.56 ± 14.92	68.15 ± 8.16	0.879
Inferior RNFL thickness (µm)	122.42 ± 19.45	118.33 ± 15.78	0.298

^*^: Data are expressed as mean ± standard deviation

FMS: Fibromyalgia syndrome; RNFL: Retinal nerve fiber layer

Correlations between disease activity and ophthalmologic measures were examined. There was no significant correlation between FIQ values, OCTA parameters, and RNFL thickness measures (*p* > 0.05). Additionally, no significant correlation was detected between symptom duration and ophthalmologic parameters (*p* > 0.05).

## Discussion

The investigation of rheumatic disorders using OCT and OCTA measures has gained considerable interest in recent years [19–22]. The current study investigated the ophthalmic vascular alterations in FMS and assessed the thickness of the RNFL. Within this framework, an extensive and thorough examination was conducted to quantify the density of superficial and deep vascular structures in the retina and choriocapillaris. Total retinal parafoveal thickness and foveal density were significantly higher in FMS patients. Nevertheless, there was a lack of significant disparity between the FMS and control groups in the majority of OCTA assessments. The measurements encompassed OCTA parameters, particularly the superficial and deep structures in several quadrants. The RNFL thickness assessments demonstrated no significant difference between the groups. Furthermore, no significant correlation was observed between FIQ values and any OCTA parameter or RNFL thickness measurement.

Most OCTA parameters, including superficial and deep vascular structures, were not significantly different from those of healthy controls in our article. Garcia-Martin et al. [23] conducted research that mainly investigated superficial vascular alterations in FMS patients. There was no discernible variation in vascular density in the macular region between healthy controls and those with FMS. Another research, which included extensive evaluations of ocular vascular structures, reported a substantial decrease in superficial vascular plexus density in the superior and inferior quadrants but no difference in the other quadrants, deep vascular structures, and choriocapillaris [10]. Urfalioglu et al. [24] assessed the blood flow region in the optic nerve head using OCTA. They categorized FMS patients based on the severity of their disease. Researchers discovered that the vascular structures of the eye were altered, particularly in those with severe FMS. Specifically, there was a tendency for the blood flow area in the optic nerve head to rise in patients with severe FMS. Sevimli et al. [25] observed a significant increase in the choroidal vascular index among FMS patients.

Several potential mechanisms underlying the concept that ocular vasculature may be affected in FMS patients. The cardiovascular system is affected in FMS patients, and the equilibrium between the sympathetic and parasympathetic nervous systems is disturbed [26, 27]. FMS is also associated with alterations in the hypothalamic-pituitary-adrenal axis, neurotransmitter, and catecholamine concentrations [28, 29]. The mechanisms mentioned above and balance disruption may affect ocular vascular perfusion. Nevertheless, our findings broadly do not support these hypotheses. Most of the OCTA parameters were similar to those of healthy controls. Furthermore, no notable correlation was found between the severity of the disease and the OCTA characteristics. Furthermore, the research in the literature lacks consistency. There might be additional factors contributing to this. Initially, it should be noted that sample sizes are restricted. The symptom duration, drug use, non-pharmacological treatment approaches such as exercise, and the presence of comorbid conditions may influence the outcomes. The investigations did not assess the autonomic nervous system condition, the balance between the sympathetic and parasympathetic systems, and levels of catecholamines and neurotransmitters in FMS patients. Alterations in these factors may have influenced the outcomes.

Our study found no statistically significant difference between the groups’ evaluations of RNFL thickness. In addition, no significant correlation existed between FIQ scores and RNFL thickness measurement. Sevimli et al. [25] observed a reduction in RNFL thickness only in the superior quadrant. There was no difference with controls in other quadrants. Boquete et al. [30] reported a decrease only in the inner inferior quadrant. However, Urfalioglu et al. [24] found decreased RNFL thickness in diffuse and various quadrants. The outcomes of our study do not provide evidence to support the hypothesis that neurodegeneration and decreased RNFL thickness play a role in the etiopathology of FMS.

Several limitations should be addressed when interpreting these findings. First, the study’s cross-sectional methodology limits drawing any causal conclusions about the association between FMS and alterations in the retinal vasculature and RNFL. Longitudinal investigations are needed to determine temporal associations and causality. Second, the sample size was limited, which may have reduced statistical power to detect differences in some parameters and increased the likelihood of type II errors. Research with larger sample sizes is required to validate these findings and identify additional subtle differences. Furthermore, to prevent confounding effects, the study excluded individuals taking FMS-related drugs; nonetheless, this exclusion criterion may have reduced the generalizability of the findings to all FMS patients, particularly those on therapy. The state of the autonomic nervous system and sympathetic-parasympathetic nervous system balance were not assessed in the participants. The majority of participants were female.

## Conclusion

Although there were slight differences in retinal parafoveal thickness and foveal density, the comprehensive results indicated that the eyes of FMS patients did not display substantial neurovascular or neurodegenerative alterations compared to those of healthy individuals. Although it was hypothesized that FMS, a disorder characterized by probable neurovascular abnormalities, may cause noticeable alterations in ocular structures, our data did not substantially support this notion. These findings indicate that the anticipated neurovascular and neurodegenerative alterations in the eyes of individuals with FMS may not be as significant as initially thought. The lack of substantial disparities in most OCTA metrics and RNFL thickness does not provide evidence to suggest that FMS significantly impacts these particular ocular structures. To gain a more thorough understanding of the ocular consequences of FMS, longitudinal investigations with a larger sample size are necessary. These studies should include extensive evaluations of autonomic nervous system function and other aspects potentially influencing the results.

### Electronic supplementary material

Below is the link to the electronic supplementary material.

Supplementary Material 1

Supplementary Material 2

Supplementary Material 3

Supplementary Material 4

Supplementary Material 5

Supplementary Material 6

Supplementary Material 7

Supplementary Material 8

## Data Availability

Raw data can be shared if requested.
